# Big hearts, small hands: a focus group study exploring parental food portion behaviours

**DOI:** 10.1186/s12889-017-4711-z

**Published:** 2017-09-18

**Authors:** Kristina Curtis, Louise Atkins, Katherine Brown

**Affiliations:** 1 Behaviour & Interventions Research, Faculty of Health & Life Sciences, Coventry University (Joint with Public Health Warwickshire), Mile Lane, Coventry, CV1 2NL UK; 2 UCL Centre for Behaviour Change, University College London, London, UK

**Keywords:** Childhood weight management, Childhood obesity prevention, Parental dietary behaviours, Health promotion, Behaviour change, Theory, Portion sizes

## Abstract

**Background:**

The development of healthy food portion sizes among families is deemed critical to childhood weight management; yet little is known about the interacting factors influencing parents’ portion control behaviours. This study aimed to use two synergistic theoretical models of behaviour: the COM-B model (Capability, Opportunity, Motivation – Behaviour) and Theoretical Domains Framework (TDF) to identify a broad spectrum of theoretically derived influences on parents’ portion control behaviours including examination of affective and habitual influences often excluded from prevailing theories of behaviour change.

**Methods:**

Six focus groups exploring family weight management comprised of one with caseworkers (*n* = 4), four with parents of overweight children (*n* = 14) and one with parents of healthy weight children (*n* = 8). A thematic analysis was performed across the dataset where the TDF/COM-B were used as coding frameworks.

**Results:**

To achieve the target behaviour, the behavioural analysis revealed the need for eliciting change in all three COM-B domains and nine associated TDF domains. Findings suggest parents’ internal processes such as their emotional responses, habits and beliefs, along with social influences from partners and grandparents, and environmental influences relating to items such as household objects, interact to influence portion size behaviours within the home environment.

**Conclusion:**

This is the first study underpinned by COM-B/TDF frameworks applied to childhood weight management and provides new targets for intervention development and the opportunity for future research to explore the mediating and moderating effects of these variables on one another.

## Background

It has been widely contended that the most prevalent category of determinants and risk factors for childhood obesity (e.g. dietary behaviour, physical activity and sedentary behaviour), begin within the family environment [1] where children consume around two-thirds of their daily food intake [2]. Children are dependent on their parents and carers to provide food that is conducive to both a healthy weight and development [3]. Parents exert strong influences on children’s weight status through an array of mediators including: availability of food, meal structure, their own weight status, socialisation of food practices, food preferences, socioeconomic status, attitudes towards their children, family structure, and cultural practices [1]. Hence, family-based approaches are now well recognised in the childhood weight management literature, where they are considered the ‘gold standard’ for improving children’s weight status and overall health [4]. Despite this there has been a lack of understanding regarding exact parental influences on children’s dietary behaviours within the context of the obesogenic environment [5] and consequently, how to directly target parents in weight management interventions [6].

Theory is helpful in understanding behaviours as a first step in intervention development [7] and there are a number of theoretical frameworks that can be applied to childhood weight management. However, these frameworks are not without their limitations and so far, one theory has not been shown to be more effective for developing interventions for weight management over another [8]. Prevailing health behaviour change theories in Health Psychology comprise social cognitive theories which rely heavily on individual reflective cognitive processes and largely ignore automatic processes comprising of emotional variables, impulses, habits, associative learning and self-control [9]. They primarily focus on intra-individual factors as opposed to wider social and environmental factors [10], therefore, they can only weakly address the parent-child dyad and the environmental system processes where interactions among family members impact on parents’ behaviours [5]. Furthermore, principal theories of behaviour change also fail to address the full canvas of relevant theoretical constructs for behaviour change where there is significant overlap between constructs [11, 12]. These theoretical shortcomings have led to a response for the need for an overarching holistic theoretical framework where experts in areas of health psychology, theory and health services have identified 128 initial theoretical constructs drawn from 33 psychological theories [13]. Key constructs were then grouped into 12 (recently refined to 14) theoretical domains such as ‘Knowledge’, ‘Skills’ and ‘Emotion’ that resulted in the ‘Theoretical Domains Framework’ (TDF) and function as mediators of behaviour change [14, 15]. The TDF is designed to be ‘an inclusive, rather than selective, approach to exploratory research in the field of implementation’ [14].

Another approach that aims to support the translation of theory into practice is the COM-B model, which was developed to counteract the inability of the majority of prevailing theories to provide strategies to change behaviour, and as part of a ‘method for characterising interventions and linking them to an analysis of the targeted behaviour’ [7:1]. It is essentially a behavioural system that posits the interaction of three components: Capability, Opportunity and Motivation (COM) which result in the performance of Behaviour (B) [16] (See further explanation in [17]). The COM-B model bridges the gap left by many of the social cognitive and ecological models that fail to account for automatic processes such as impulses and emotions along with neglecting ‘factors as a system level’ (e.g. HBM) [16].

**Fig. 1:**
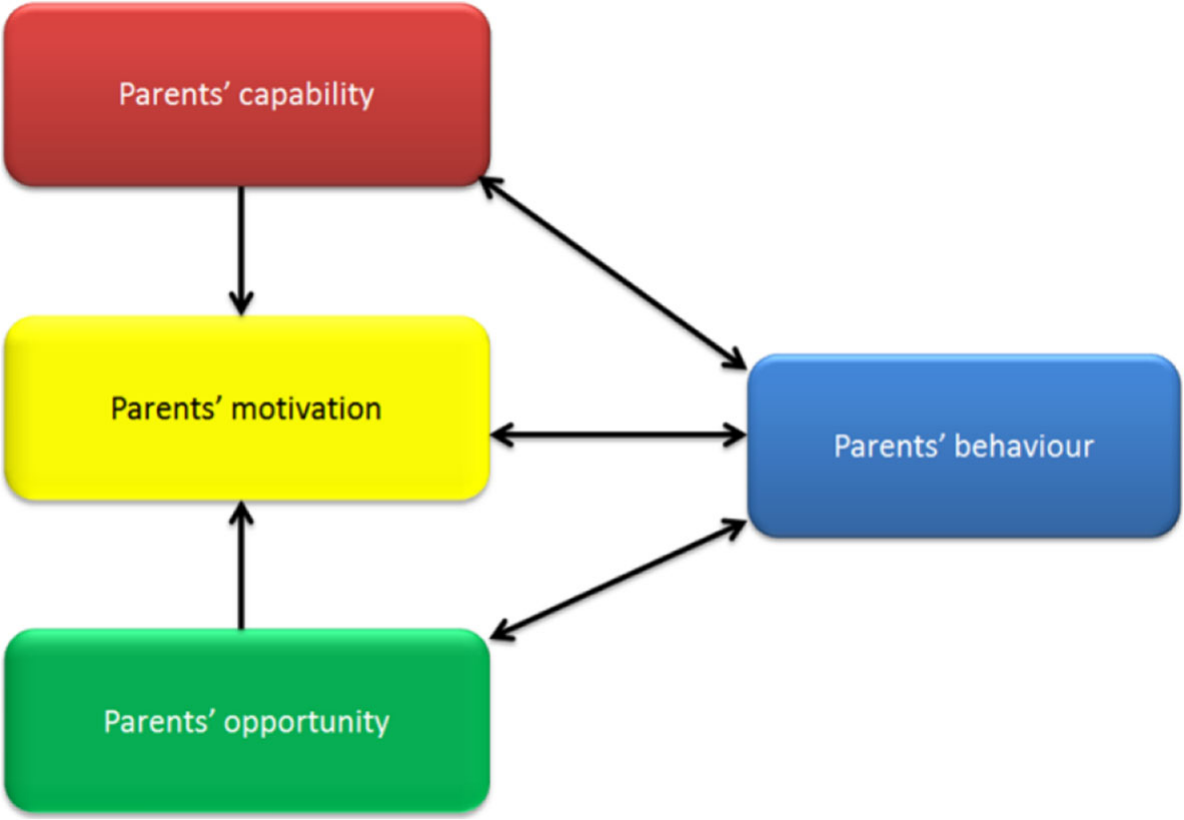
Application of the COM-B model to parents’ behaviour

**Fig. 2:**
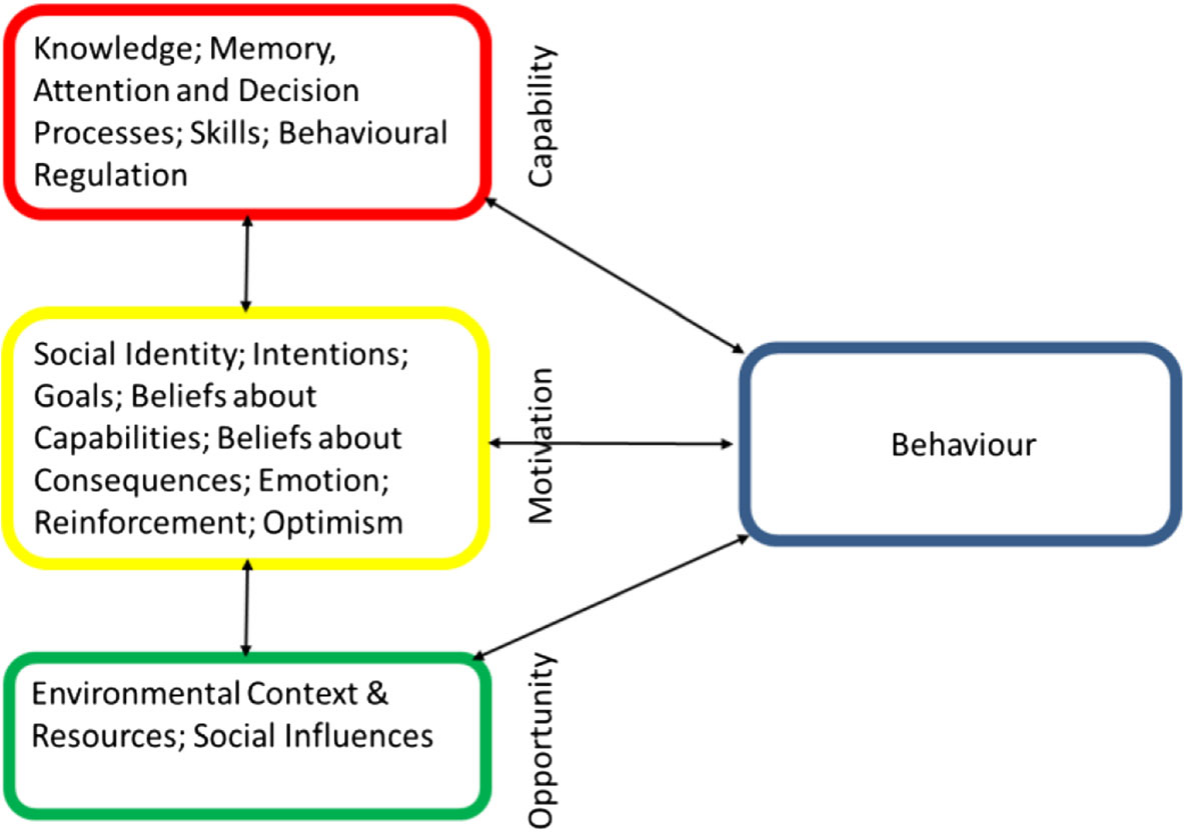
Theoretical constructs of the TDF grouped into the COM-B model

Figure 1 illustrates the relationship between the various COM-B components [7]. For example, eliciting positive changes in a person’s capability or opportunity can potentially increase a person’s motivation to perform a behaviour, whereas motivation can only increase opportunity or capability through performing a behaviour itself [18]. Further work has now grouped the theoretical constructs of the TDF into the COM-B model (see Fig. 2), allowing researchers to use the TDF in a way that postulates links between the domains [18].

This allows intervention designers to start with a COM-B analysis of the problem before drilling down into greater detail using the TDF, and ensures that the C O and M components and their TDF subdivisions can be linked to the Behaviour Change Wheel (BCW) intervention design method [18]. The BCW helps to overcome limitations of previous theories of behaviour change which do not specify how exactly they should be used to bring about change in a behaviour [8, 16]. In contrast, the BCW allows its theoretical conditions to be operationalized for changing behaviour and provides direct strategies for achieving this. This is particularly important for specifying in behaviour change protocols, where the link between theory and practice is not always explicit making it difficult to identify which theory, if any, has guided the intervention approach [11]. The difficulty in recognising theory is exacerbated in childhood weight management interventions as a result of heterogeneity that has limited researchers’ ability to link interventions to weight outcomes [19].

The current study was conducted in response to the need for a broader understanding of the range of factors influencing parents’ dietary behaviours with their children. Following the steps in the BCW led to the selection of the target behaviour: food portion size regulation (refer to [17] for more detailed information on how the target behaviour selection process). Target behaviour selection involves consideration of a range of potential behaviours that can be changed to address the health problem, and consideration of relevant evidence in making that decision. In a review of childhood weight management interventions [20], regular large portions of high density calorie foods and sugary drinks were reported to increase the risk of childhood overweight and obesity. This is not too surprising considering the food industry within western industrialised nations provide enormous variety and accessibility of cheap high energy foods (including fast foods) and increased food portion sizes, creating a demand where consumers want larger quantities of foods for lower prices [21–23]. Subsequently, it has been argued that this change in our food environment has contributed significantly to the obesogenic environment [24, 25] and parallels the rise in both childhood and adult obesity [23, 26, 27]. It has also been demonstrated that portion sizes have increased in home cooking [28] and yet so far, limited research has been conducted with parents to understand the influences on their portion control behaviours. Since conducting the study, it should be noted that the World Health Organization (WHO) has recognised that the development of healthy food portion sizes among families is deemed critical to childhood weight management [29]. A qualitative research design was necessary for identifying relevant theoretical domains that potentially influence the target behaviour. The study also takes account of a systematic review of previous qualitative research in this area revealing that the majority of the studies were: based in the United States, Australia and Canada (only one was UK based); included parents of children less than five years old (62%); included parents who may or may not have had overweight children; did not use theoretical frameworks or conceptual models to guide research and analysis and; explored a range of weight related behaviours including exercise [30] making it difficult to understand which sources of influences on behaviours led to certain outcomes [31]. Furthermore, there is a paucity of research investigating parents’ emotional barriers to childhood weight management, where most of the research has focused instead on children’s emotional barriers (e.g. [32, 33]). To bridge these gaps, this study represents the first to conduct research with a UK population of parents with mainly overweight children all above the age of five; guided by two theoretical tools: the COM-B model and TDF and; focusing on one main nutrition behaviour: parental provision of age appropriate portion sizes. The results of the study have been summarised in a separate paper used to inform the development of a mobile health app for local public health services supporting parents in portion control (See [17]). However, the former paper focused on the development process and not the theoretical findings. Therefore, this paper aims to explore parents’ capability (C), opportunity (O) and motivation (M) towards portion control behaviours (B) with their children and the greater level of detail within these components afforded by using the TDF.

## Methods

### Study aim and design

Two theoretical frameworks, the COM-B model and TDF, were used to deductively identify influences on parental dietary behaviours with a focus on portion control in the context of childhood weight management. Prior to recruiting parents, a focus group was conducted with all active family weight management case workers operating in the local area as a way of scoping the problem and allowing: (i) familiarisation with the context of childhood overweight and obesity; and (ii) a ‘preliminary theoretical explanation’ to help guide decisions regarding which TDF domains to focus on with parents. The remaining five focus groups comprised of parents (ranging from between three and eight participants in each) and were conducted to identify targets for the intervention until themes based on parents’ responses achieved saturation. Focus groups facilitated interaction among participants that stimulated rich data for analysis [34] where the researcher, KC, played an active role in guiding the discussions for data collection [35]. These group processes helped participants to exchange and clarify their ideas and experiences in ways not usually possible in a one-to-one interview [36]. Open-ended, semi-structured questions structured around relevant TDF domains (Table 1) allowed for in-depth responses around issues that were important to participants using their own terminology and language [37].

**Table 1:** COM-B Analysis of parents’ portion control behaviours

COM-B	TDF	Topic schedule
Psychological capability	*Knowledge*	Parents' knowledge of appropriate portion sizes for children’s meals Parents’ knowledge of what children’s daily food intake should be (how do they judge what is enough/too much*?)*
Psychological capability	*Cognitive and interpersonal skills*	Parents’ skills in talking to their children as well as family members about making changes to their portion sizes
Psychological capability	*Memory, Attention and Decision Making Processes*	Parents’ awareness of portion size guidance
Psychological capability	*Behavioural Regulation*	Parents’ monitoring of children’s portion sizes (includes size of portions and frequency of consumption)
Psychological Opportunity	*Environmental Context and resources*	Objects in parents’ environment to assess portion sizes
Social Opportunity	*Social Influences*	Other people in parents’ environment that either hinder or enable age appropriate portion sizes
Reflective Motivation	*Beliefs about capabilities*	Parents’ beliefs about capabilities in reducing children’s portion sizes.
Reflective motivation	*Beliefs about consequences*	Parents’ beliefs about the consequences of childhood overweight
Automatic motivation	*Emotion*	Parents’ emotions related to portion control
Automatic motivation	*Reinforcement*	Parents’ eating habits
N/A	N/A	Are there any other barriers to portion control within the home environment that we haven’t already talked about?

### Participants and recruitment

A ‘theoretical’ and ‘purposive’ sampling approach was used to recruit participants for the study as opposed to a randomised approach which is more suited to quantitative inquiries [37:110]. Eligible participants included three sub-groups of participants to allow for triangulation of data [38]. Participants comprised: (1) family weight management case workers working with families with overweight children, (2) parents with overweight and or very overweight children and (3) parents with healthy weight children ≥5 years. This supported the convergence between multiple sources of data to generate themes, validate findings, improve credibility and acquire greater overall understanding of the phenomena [39]. Participants were recruited through emails distributed to the local public health department, community family weight management groups and a university. Further details of the study sample and methods have been previously described (see [17]). Ethical approval for focus groups was obtained from the University of Warwick Biomedical and Scientific Research Ethics Sub-Committee in advance of the research being undertaken.

### Sample characteristics

The family weight management case workers (*n* = 4) were all females (FG1). Participants recruited from the weight management programmes (*n* = 14) (FG2, FG3, FG4, FG5) comprised of females (*n* = 12) and males (*n* = 2) and had children classified as very overweight (53%), overweight (33%) and healthy weight (7%). Parents with healthy weight children (*n* = 8) recruited from the university (FG6), were academics (*n* = 3) and office administrators (*n* = 5) and included female (*n* = 6) and male (*n* = 2) participants.

### Procedures and setting

Six focus groups with case workers (*n* = 4), parents with overweight children (*n* = 14) and parents with healthy weight children (*n* = 8) took place at university and community settings. All focus groups were facilitated by one moderator. Consent forms were administered and signed before the focus groups began. Krueger and Casey’s (2000) [40] principles for conducting a focus group were followed and the conversation was guided by the schedule of questions. The focus group with caseworkers took place at the university and lasted 120 min, while focus groups with parents took place at the local weight management programmes and the university, lasting 60 min.

### Data analysis

With participants’ permission, focus groups were audio recorded and transcribed verbatim and the raw data was coded using a thematic analysis. Thematic analysis involves a systematic process for interpreting data where patterns are identified and analysed to provide ‘illuminating descriptions of the phenomenon’ [41:54]. Braun & Clarke’s (2006) [41] six stages of analyses was used to explore the data. Braun and Clarke (2006) argue that thematic analysis offers ‘an accessible and theoretically flexible approach to analysing qualitative data’ [42:77]. However, the authors state that it is paramount that the theoretical framework chosen aligns with the research aims and that decisions in the process are acknowledged. Therefore, in this study, the COM-B and TDF frameworks were chosen as an overarching theoretical framework that allowed assessment of behaviour change components that were modifiable to support parental dietary behaviours. Data review and interpretation involved deductive coding using the COM-B and TDF as coding frameworks.

To ensure the ‘retest reliability’ of the analysis, audio recordings, transcripts and notes on the researcher’s thoughts while transcribing were all collected along with providing a detailed account of the data analysis process. NVivo software was used to facilitate the coding of data from the focus group transcripts [37]. In addition, the reliability of the qualitative data was further enriched by the use of an additional trained qualitative researcher who was familiar with the BCW framework and TDF framework, and who independently coded 10% of the data in order to establish inter-rater reliability. An agreement of 10/12 TDF domains was established where upon discussion, full agreement was reached. An inter-rater reliability of .83 is generally considered to be an acceptable rate [42].

## Results

### Overview

Application of the BCW framework requires identification of components of the COM-B behavioural system that need to change in order to achieve the target behaviour [7]. The findings provide an understanding of how the COM components vary according to the behaviour (regulation of portion sizes), population (parents) and context (childhood weight management) (18). All COM-B components (except physical capability) were identified as important for supporting parents in achieving the target behaviour. These components aligned with nine TDF domains as follows: *Psychological Capability*: Knowledge; Memory; Attention and Decision Making Processes; Skills; *Automatic Motivation*: Emotion; Reinforcement; *Reflective Motivation*: Beliefs about capabilities; Beliefs about consequences; Social identity; *Physical opportunity*: Environmental context & resources and *Social Opportunity:* Social influences.

### Psychological capability

#### Knowledge

The TDF defines knowledge as ‘an awareness of the existence of something’ [15:13]. Focus group discussions revealed parents lacked knowledge about appropriate portion sizes, nutritional value of food groups, and strategies for portion control.

There was consensus among parents regarding their lack of knowledge of appropriate adult and child portion sizes.

*Until I came here, I didn't really know much about portion sizes at all. (*Parent, FG4).

Responses also highlighted that parents did not typically pay much attention to the management of portion sizes and were unaware of any guidance.

*I don’t think there is any guidance for portion sizes, I mean until you mentioned it and I felt oh actually yeah I think that's an issue with our house. I don’t think we’d ever really thought about it* (Parent, FG5).

Case workers cited that often parents are not aware to what extent certain foods and beverages are ‘unhealthy’. For example, it emerged that parents were often unaware of the amount of sugar and/or fat in some foods and beverages.

*I just don’t think they understand how much one chocolate bar actually is or how much fat is actually in a portion of chips, it’s very hard to try and get that across I think.* (Case worker, FG1).

*I won't buy that again. I am surprised, Ribena had as much sugar in it as Coke and I had never known that.* (Parent, FG4).

#### Skills

The TDF defines skills as ‘an ability or proficiency acquired through practice’ [15:13]. *Skills* were identified in relation to parental ability, competence and procedural knowledge for measuring appropriate food portions and their interpersonal skills for discussing dietary and weight issues with their children.

In some instances, parents described their difficulty in assessing the quantity of servings specified in metric measurements during cooking.

*Yeah it’s also difficult to assess the portion size. If you make something how do you know whether its 80 grams or a 100 grams?* (Parent, FG5).

Some parents and case workers agreed that parents tended to use their own portion sizes of food as a guide for measuring their children’s portion sizes. Consequently, they may not differentiate between adult and child portions.

*For me, I find it particularly difficult dishing out the correct portion size for children and for adults, I suppose. I just tend to give everybody the same amount* (FG3, parent).

Across all focus groups, parents recalled their struggle in communicating with their children around dietary issues. For example, several parents expressed their difficulty in helping their children understand the reason why they should have a smaller portion of food compared to adult portions.

*Yeah, I think if you give them a smaller portion size, then [child’s name] will say why have I got less and doesn't really understand that she's got a smaller body and needs less than adults do* (Parent, FG3).

In addition, some case workers and parents felt that it was more challenging for parents to discuss dietary and weight related issues with older children. Typically, this challenge coincided with their fears of causing their children to have anxieties about their weight (see *Emotion).*

*Yeah..I was going to say that happens quite regularly but also when you’re contacting year 6 parents where the young people are overweight..the parents don’t necessarily want to bring up the subject because of their age* (Caseworker, FG1).

### Physical opportunity

#### Environmental context and resources

The TDF defines environmental context and resources as ‘any circumstance of a person’s situation or environment that discourages or encourages the development of skills and abilities, independence, social competence, and adaptive behaviour’ [15:14]. Within this theme, focus group discussion content related to resources parents used for measuring portions.

There was a common preference among parents for using house-hold objects such as plates, spoons or bowls to measure portion sizes instead of using scales.

*I get a cup or even pasta, put in a cup first so I know that, that's going to be enough for me if I were doing it for myself or if I’m doing it for like three of us I’ll put a whole cup in* (Parents, FG5).

However, while household objects such as a spoon or a cup were described as a facilitator for measuring food portions, adult sized plates and bowls were described as a hindrance for measuring appropriate child food portion sizes.

*.. my daughter has gone to a larger plate as she got older. When it gets to a larger plate then that’s when it gets it bit out of hand* (Parent, FG5).

One mother shared her experience of losing a significant amount of weight using half the plate to measure her portions, highlighting the excessive sizes of household plates.

*I mean I've just lost recently 4 stone.. Yeah, and that is by eating off half the plate*. (Parent, FG3).

### Social opportunity

#### Social influences

Social influences can be defined as ‘interpersonal processes that can cause individuals to change their thoughts, feelings, or behaviours’ [15:14]. Focus group discussions revealed that prevalent social influences impacted both the frequency and size of portions within the home environment. These encompassed partners and grandparents.

Case workers described how grandparents in particular, can make it difficult for parents to ensure their child is eating healthily and that they may ‘undo’ parent’s good work.

*mmm..and on a positive notes..um..some of my families..they are really trying to make this change but Grandma..they go over to Grandma’s and Grandma is giving them ALL THIS STUFF!* (Case worker, FG1).

In addition, it was evident from focus group discussions with parents and case workers that some families are dependent on children’s grandparents for child care. In this regard, several parents recalled their fear of causing conflict with grandparents over food issues.

*It’s got to the point now that’s there’s no point because it will just cause an argument…Because you can sort of keep them away from..just send them to Nan’s when they can have a treat and I think that’s the easy option. (*Parent, FG2).

Partners were also identified as an important barrier to providing healthier food options and appropriate portion sizes for their children.

*And this is what my issue with my husband giving them far too much is that he is using pasta bowls that's adult size deep dish bowls that go on forever. I need to stop him from doing that.* (Parent, FG5).

Several mothers in the focus groups described their frustrations with their partner’s failure to support them in making changes. This highlights the need to involve the whole family in making changes to eating habits and not just the individual child.

### Automatic motivation

#### Reinforcement

Reinforcement can be defined as ‘increasing the probability of a response by arranging a dependent relationship, or contingency, between the response and a given stimulus’ [15:13]. Within this theme parental eating habits appeared to reinforce parents’ dietary behaviours with their children.

Focus group discussions revealed how parental eating habits and food preferences may act as barrier to changing their dietary behaviours with their children. From case workers perspectives, greater difficultly for children arises when their parents are not simultaneously changing their own eating habits.

*I’ve got families as well where the child’s really trying and they are you know..14 ½ stone but Mum’s still got the ‘Clover’ you know..so he’s trying but she’s still buying the full fat things..you know..so it’s hard for them* (Case worker, FG1).

There was agreement among some parents that their own eating habits influenced their children’s eating preferences. Parents were less likely to provide food for their children that they disliked themselves.

*Yeah I think also when planning it’s also by your own eating habits. So if you don’t like vegetables then you will be less likely to cook vegetables for your kids* (Parent, FG3).

#### Emotion

The TDF defines emotion as ‘a complex reaction pattern, involving experiential, behavioural and physiological elements, by which the individual attempts to deal with a personally significant matter or event’ [15:14]. Childhood overweight is a highly emotive issue for parents. Data provided insight into a number of emotional barriers, including parental fear, guilt, and denial. These emotions may directly or indirectly impact on their dietary behaviours with their children.

Focus group discussions repeatedly underscored parents’ fears of causing their child to feel anxious about their weight if they attempted to discuss it with them.

*We might try and tackle it a little bit, try discuss it with him..but we don't want him to go the other way and you know..have anxieties about that* (Parent, FG3).

There was agreement among case workers that parents feared the onset of eating disorders if they share results from the UK Government’s surveillance programme (informing parents of their children’s weight status) with their children.

*So..It’s such a sensitive subject isn’t it? And um.. a lot of families don’s want to share the results with the children because they worry about the effects its going to have on the child..whether their going to become anorexic or whether they become bulimic.* (Case worker, FG1).

Parental emotions towards their children’s weight gain appeared to be heightened through parents’ own feelings and experiences of being overweight as children.

*I mean sometimes the mum will say “oh I’ve tried in the past to lose weight..” ..what’s quite interesting is that you’re on the phone with them and obviously you’ve struck a chord with them cos they say when they were young..and one mum said “oh well I hope you’re not going to say what my mum said to me when I was young”* (Case worker, FG1).

Other types of fears that emerged related to parents’ fear of the negative effects of restricting children’s snacks and/or encouraging healthier eating behaviours (which also overlaps with *beliefs about consequences*).

*I think those issues around parents wanting to be liked by their children, is another problem I have. So if they say they don’t like something or they don’t want to make them unhappy or stressed by forcing them to eat stuff* (Parent, FG2).

Case workers agreed that parents may fail to recognise their children’s weight because they are in denial, suggesting an emotional barrier may prevent parents from accepting the problem.

*I think..they don’t actually admit they’re overweight..they just say I’m overweight, they’ve got my build..bit of denial* (Case worker, FG1).

*I wouldn’t have said my son is overweight* (Parent, FG2).

Parents also tended to blame their children’s weight gain on external factors such as schools and the food industry; reflecting their reluctance to take responsibility for their children’s weight.

*I would say it’s more like school and the peer pressure that I was saying earlier about how it affects things and also the after school club don’t offer healthy snacks.* (Parents, FG5).

### Reflective motivation

#### Beliefs about capabilities

Beliefs about capabilities are defined as ‘acceptance of the truth, reality, or validity about an ability, talent, or facility that a person can put to constructive use’[15:13]. Focus group discussions relating to this theme centred on parental confidence in making changes to their children’s diets and weight status and their own experiences of trying to manage their weight.

Some parents with overweight children admitted that they had low confidence in their ability to make changes to their children’s diet.

*M: And what are your thoughts on how confident you feel towards changing your children’s eating habits?*

*P3: I’d give in too easily.*

*P8: Not very confident.* (Parents, FG4).

Case workers believed that parents’ lack of confidence may stem, in part, from their own unsuccessful attempts at losing weight, which also represents an *emotional* barrier.

*I think if you’ve got..I mean I’ve spoken to mums and they’ve still tried to lose weight in the past and tried every diet going so they’ve tried it all for themselves. How will are they to try it for their child?* (Case worker, FG1).

This highlights the importance of interventions supporting parents’ confidence in managing both their own weight and their child’s weight, as echoed by case workers’ comments below.

*Self-esteem I think is..if you can encourage somebody to increase their self-esteem, then their willingness to make any sort of change grows quite rapidly doesn’t it?* (Case worker, FG1).

#### Beliefs about consequences

Beliefs about consequences is defined in the TDF as the ‘acceptance of the truth, reality, or validity about outcomes of a behaviour in a given situation’ [15:13]. Focus group discussions concentrated predominantly on parental beliefs around: the consequences of measuring food portions and; the consequence of overfeeding from cooking excess food. Furthermore, an indirect impact on the target behaviour appeared to be parental beliefs around the consequences of being overweight as a child and adult.

Some parents held the belief that measuring appropriate portion sizes, as recommended on food packaging, requires too much time.

*I still don’t have the time to figure out..I just don’t want to* (Parent, FG2).

It was evident from focus groups with parents that the majority of parents held the belief that if they cooked too much food, they will overfeed. There was strong agreement among parents that this was due to their preference of not wasting food.

*So if you’ve over cooked, you will overfeed…. I don’t like to throw it in the bin the so it goes on the plate (Parent, FG4).*

Parental beliefs around the consequences of being overweight (as children and/or adult) may indirectly impact on parental reflective motivation towards changing their own and their families’ dietary behaviours. Case workers described parents’ difficulty in linking adult overweight with health problems.

*P3: I’ve got one family where mom’s got diabetes, dad’s dad died of a heart attack really young, his brother had died really young..but they couldn’t relate that to any sort of..*

*M: being overweight*

*P3: No and they couldn’t even see that it was something they could make preventative measures towards her* (Case worker, FG1).

In addition, case workers agreed that part of this difficulty that some parents have in understanding the impact of their behaviour on later health problems is because they prioritise the present over the future.

*I think it’s also difficult for some families to anticipate the future isn’t it..so if they’re living very much day to day, week to week, explaining to them that the health implications for their future aren’t good isn’t always something that they can relate to* (Case worker, FG1).

This was reflected by the majority of parents across all parental focus groups who were most concerned with their child being teased as a consequence of being overweight.

*I think for me it’s the teasing, you know, the peer pressure because I was teased really badly at school for being overweight and that's my main memory of secondary school just being told you’re fat and you know and I don’t want them to go through that* (Parent, FG3).

Case workers agreed that some parents perceived their children’s weight as inevitable due to parental overweight.

*Often they say because they’ve got my build so because the parents are overweight they just accept the fact that the child’s going to be overweight..I think..they don’t actually admit they’re overweight..they just say i’m overweight, they’ve got my build..bit of denial* (Case worker, FG1).

## Discussion

### Summary of findings

Findings suggest that parents’ internal processes such as their knowledge and skills (Capability), emotional responses, habits and beliefs (Motivation), along with social influences from partners and grandparents, and environmental influences (Opportunity) relating to items such as household objects, all interact to influence portion size behaviours within the home environment.

### Parents’ capability

Parents expressed difficulty in quantifying portion sizes using metric measurements which supports other research [43]. Furthermore, parents and caseworkers also highlighted parents’ difficulties in their attempts to discuss food and weight issues with their children which builds on previous research showing that greater BMI is associated with poor parent-child communication (5). In addition, Sealy et al., (2012) [44] reported parental frustrations with the poor level of family communication around overweight and obesity. Sealy et al., (2012) also found that parents expressed the need for specific information around ways to talk to their children in relation to weight and nutrition without harassing them or lowering their confidence. This highlights emotional aspects of parent-child communication. Parents in the current study were concerned about lowering their children’s confidence and or/evoking anxiety in their children, particularly older children.

#### Parents’ reflective motivation

The findings indicated that some parents believed measuring appropriate portion sizes requires too much mental effort which is supported in previous research with consumers also comprising of mainly female participants [45]. In contrast, Slater et al., (2010) found that parents are least likely to report this as a barrier to making changes to their dietary behaviours [46]. Although, it should be noted the survey tool used in Slater et al’s., (2012) research, did not differentiate between healthy eating behaviours.

Focus group data highlighted parents concern towards wasting food which is reflected in the extant literature [45, 47, 48]. Indeed, research suggests that parents’ core values in relation to food waste are often instilled during their own childhood, where it was often expected that all food on the plate should be eaten [43]. In this regard, parents may benefit from increasing their skill in cooking appropriate amounts of food to prevent serving too much food on plates.

The majority of parents across all focus groups indicated that they were most concerned with the risk of their child being teased as a consequence of being overweight. Arguably, parental concerns towards weight-related teasing are justified when we take into account that appearance-related teasing is the most widespread among children, and overweight children experience higher levels than healthy weight children [49, 50]. Similar findings were reported in a systematic review [30] where parents perceived overweight and obesity as issues for the future and were more concerned with cosmetic appearances compared to health consequences which is further supported in other qualitative research with parents (see [51, 52]). These findings support recommendations that interventions need to steer away from disease related messages such as linking diet to heart disease, and focus more on immediate consequences such as bullying, asthma, dental health and school performance [53].

### Parents’ motivation

Parents conveyed their habit of using house hold objects for measuring portions in the home environment, a preference well documented in consumer research (e.g. [54, 55]). In the current study, outsized dinnerware was identified as a barrier to providing appropriate portion sizes. Arguably, the size and shape of dinnerware, glasses and utensils may act as a prime to consumption responses [56]. Previous research has shown that larger dinnerware leads to serving and consuming larger portions [57, 58], as explained by the Delboeuf illusion (see. [59]). The findings are in line with previous research suggesting that eating behaviours are influenced by the environment resulting in automatic eating decisions, and that people are often unaware that the environment is affecting their eating behaviour [60]. It has been proposed that habits override ‘conscious action control and automatically maintain dietary behaviors’ [57:11]. In this regard, according to the authors, overcoming habits require effort, which may be influenced by a number of internal and external influences including parents’ self-efficacy and perceived lack of time to make changes, making it difficult to override these habits. However, according to Spence et al. (2013), implementing family-based interventions that encourage good portion size practices from an early age may help to break these habits. This is particularly important in light of research showing that portion size habits become ingrained from mothers’ portion size practices [43].

The current research validates previous research showing that parents often serve and provide food based on their own food preferences [61]. Research suggests that children’s ability to self-regulate is linked to parental eating habits in regards to modelling of out of control eating and dieting [5, 61, 62]. This provides a rationale for interventions to encourage parents, to change their own diets and adopt a ‘do as I do, not what I say’ approach [64:270]. In agreement with Fassihi, et al., (2012), interventions need to be more effectively tailored to parents who are overweight to help them change their own weight related behaviour as a way to support children [63].

An important limitation previously acknowledged with some of the prevailing theories of behaviour change in health psychology is their failure to account for the emotional factors that influence behaviour. It was evident in the present study that parents experience a range of emotions that may impact their management of their children’s eating habits. Despite this, the majority of research has focused instead on children’s emotional barriers (see [32, 33, 64]). While the current findings support existing evidence on emotional influences relating to parents’ fear of eating disorders [53, 65], guilt of restricting food [66], and fatalism [67] they also add a number of new insights including parents’ fear of being disliked by their children and causing familiar conflict with Grandparents, especially when they rely on them for childcare. Furthermore, while parents’ denial of their child’s overweight has been cited in the literature [52], a theoretical analysis of focus group discussions helped to delve deeper into this emotion and suggested several potential contributory factors that may give rise to parental denial including; parents’ own overweight status (which parents may not wish to address); parents’ view that their children’s weight gain is inevitable because it persists within the family and; parents’ own experiences of unsuccessfully losing weight which appeared to lower their confidence in being able to help their children. In this regard, we can see why it may be easier for parents to blame external factors for their children’s weight gain [52]. Arguably, a more thorough understanding of parents’ emotional barriers provides further opportunities for resolving them.

### Parents’ social opportunity

Grandparents were cited by both parents and caseworkers as barriers to parents’ regulation of their children’s frequency of food portions where grandparents often provide children with supplementary food. Previous research cites grandparents as important influencers on families’ food consumption [53, 68]. According to Faith et al., (2012), grandparents can influence the home food environment, attitudes and family values [57]. The current research builds on knowledge in this area and reveals parents’ ambivalent emotional responses involving both frustration towards grandparents behaviour combined with fear of causing familial conflict particularly where parents depend on their parents for child care. Mothers also expressed frustrations with their partners providing children with greater portion sizes than needed. This is an important influence on children’s consumption behaviour; particularly given previous research highlighting fathers who are unsupportive of healthier eating habits have children with higher BMIs [69].

It is also important to consider that previous theoretical accounts such as the TPB and HBM, fail to take account of the full environmental influences on behaviour (opportunity) that include not only the environmental context and resources but also the social environment. For example, the current research highlighted that parents limited time to make changes to their dietary habits with their children and their tight fiscal situation resulted in food economics and acquiring more food for less money. However, social influences involving grandparents and partners’ provision of larger portions to children, also interact with these aforementioned environmental influences and have a synergetic effect on parental management of their children’s portion sizes. Therefore, drawing on a more holistic approach to guide the research and analysis of the target behaviour allowed identification of further determinants, thus enabling more extensive ways of targeting this behaviour for change.

Other conceptual models that have integrated different theoretical approaches still fail to offer a comprehensive picture of the problem. For example, Golan and Weizman (2001), combine a behavioural, social learning, and family systems approach in their conceptual model where parents are also viewed as the agents of change [70]. Whilst Golan and Weizman’s (2001) approach does address the home environment and the importance of restructuring it to support healthier habits, it relies heavily on changing parental cognitions and increasing parenting skills without consideration of parental emotional barriers and dietary habits and how these should be overcome. In addition, similar to other approaches exploring and underpinning childhood weight management interventions (e.g. [71]), Golan and Weizman’s (2001) model targets a number of dietary habits and physical activity behaviours simultaneously and therefore lacks the detailed specificity gained from targeting one main behaviour identified as the most important within a system of behaviours relevant to the health problem. However, Golan and Weizman’s (2012) model does highlight the importance of addressing the broader family context such as parenting skills.

Brown et al., (2014) [71] have also explored the issue of childhood weight management and provide a qualitative account that supports some of the theoretical domains findings in the current research such as parental knowledge, emotion, beliefs about capabilities and environmental resources. However, Brown et al’s (2014) data is drawn from a non-UK population and focuses on the barriers to weight management as opposed to the context of parents’ portion behaviours. In addition, Brown et al., (2014) conducted atheoretical research limiting their ability to postulate interactions between theoretical domains. In contrast, arguably the current data can be better understood within the COM-B model’s proposition that parental capability and opportunity can influence parental motivation to carry out portion control behaviours. For example, parental skills in measuring portion sizes (psychological capability) appeared to influence their confidence in their ability to carry out this behaviour (reflective motivation), and parents’ resources for measuring portion sizes such as plates (physical opportunity) appeared to influence their portion measuring habits (automatic motivation).

### Practical implications of the findings

While there are some findings that are already established from previous research, their new classification in a COM-B/TDF approach, along with several novel findings contribute to new knowledge and together these enable a fuller picture of the range of factors that need to be considered in intervention design, and the way in which these might be simultaneously addressed within the same interventions. For example, so far efforts to explain the consumption of large portion sizes have focused mainly on the shape and size of dinnerware which provide visual cues that influence consumption beyond our conscious awareness and control in both adults and children [58, 72, 73]; the availability of low cost large quantities of high energy dense foods [21, 26, 27, 74, 75]; and genetic and biological mechanisms underlying portion size effects such as the role of reward pathways and the heritability of eating behaviours [76]. However, results in the current study show that both parents’ emotional and automatic responding (e.g. parents’ fear of being disliked by their children and portion measuring habits) and beliefs (e.g. beliefs about their capabilities to make changes their children’s diets) are also likely important influences on their portion control behaviours. Consequently, interventions targeting portion control in children focusing purely on one aspect such as environmental strategies (e.g. [72]), may not be as effective as those that also account for emotional and reflective processes associated with parental dietary behaviours with their children. Furthermore, the findings themselves also go beyond this overall relationship between COM-B domains and provide a premise for the interactions between TDF domains within the COM-B domain of Motivation. For example, both automatic processes (e.g. parental portion measuring habits, parental fear of eating disorders) and reflective processes (e.g. beliefs about their capabilities), appeared to influence parental intentions to make changes to their dietary behaviours with their children. However, previous attempts at explaining influences on parental intentions within this context have focused mainly on reflective and not automatic processes (e.g. [77, 78]). These hypotheses can now be tested using quantitative methods that will help to confirm the presence of theoretical domains and their interactions between and within COM-B domains.

### Limitations of the research

Limitations include the use of a small purposive sample, with the majority of participants being Caucasian females. Consequently, the identified views on the facilitators and barriers to parental provision of a healthier diet for their children may be less representative of fathers and male caregivers and other ethnic groups. This is also a limitation of current school based approaches that have been criticised for their failure to target spouses [79]. However, mothers engaged most on this issue as they are in most cases, the primary caregivers, as demonstrated in other research regarding childhood weight management (e.g. [51, 80]. The use of focus groups also involves limitations. There is always the potential for some participants to feel intimidated and dominated by other group members which may impede their ability to share their opinions and ideas, which may also reduce generalizability of findings [81].

### Implications for future research

Further research is required with male caregivers to explore how their experiences, thoughts and behaviours influence children’s dietary behaviours and how they can support both mothers and children. Second, the sample also comprised of mainly white British participants again limits its relevance to ethnic minorities and other nationalities, providing opportunity for further research. Third, the qualitative findings can now be used to develop a quantitative survey to help test hypotheses generated from the current qualitative findings.

## Conclusions

This is the first study to investigate parental portion behaviours with their children underpinned with the TDF/COM-B theoretical tools, for the development of an intervention for local family weight management services. Furthermore, the TDF/COM-B approaches underpin a comprehensive intervention development framework (the Behaviour Change Wheel: [18]) that supports its theoretical conditions to be operationalized to identify strategies for changing behaviour [18]. Therefore, these findings allowed work to design an intervention (See [17]) that more closely reflects real-world behaviour in a real-world context [82].

## Abbreviations

COM-B: Capability, Opportunity and Behaviour model
TDF: Theoretical Domains Framework
